# Investigating nanoplastics toxicity using advanced stem cell-based intestinal and lung *in vitro* models

**DOI:** 10.3389/ftox.2023.1112212

**Published:** 2023-01-27

**Authors:** Mathias Busch, Hugo Brouwer, Germaine Aalderink, Gerrit Bredeck, Angela A. M. Kämpfer, Roel P. F. Schins, Hans Bouwmeester

**Affiliations:** ^1^ Division of Toxicology, Wageningen University and Research, Wageningen, Netherlands; ^2^ IUF—Leibniz-Research Institute for Environmental Medicine, Duesseldorf, Germany

**Keywords:** iPSC, ASC, ESC, nanotoxicology, intestine, microplastics, particle research

## Abstract

Plastic particles in the nanometer range–called nanoplastics–are environmental contaminants with growing public health concern. As plastic particles are present in water, soil, air and food, human exposure *via* intestine and lung is unavoidable, but possible health effects are still to be elucidated. To better understand the Mode of Action of plastic particles, it is key to use experimental models that best reflect human physiology. Novel assessment methods like advanced cell models and several alternative approaches are currently used and developed in the scientific community. So far, the use of cancer cell line-based models is the standard approach regarding *in vitro* nanotoxicology. However, among the many advantages of the use of cancer cell lines, there are also disadvantages that might favor other approaches. In this review, we compare cell line-based models with stem cell-based *in vitro* models of the human intestine and lung. In the context of nanoplastics research, we highlight the advantages that come with the use of stem cells. Further, the specific challenges of testing nanoplastics *in vitro* are discussed. Although the use of stem cell-based models can be demanding, we conclude that, depending on the research question, stem cells in combination with advanced exposure strategies might be a more suitable approach than cancer cell lines when it comes to toxicological investigation of nanoplastics.

## 1 Introduction

Plastic polymers have become indispensable in our everyday life due to their favorable physicochemical properties and relatively low production costs. Annual plastics production has risen to 368 million tons in 2019 (https://plasticseurope.org) and 71% of the subsequent plastic waste ends up in aquatic or terrestrial environments (Geyer et al., 2017; de Souza Machado et al., 2018). This plastic waste is continually exposed to physical stress through abrasion or UV light and will inevitably fragment into microplastics (<5 mm) and eventually nanoplastics (<100 nm) (EFSA, 2016). In addition to environment-derived micro- and nanoplastic particles, microplastics have also intentionally been included in consumer products and can subsequently fragment to the nanoscale size (Enfrin et al., 2020). Other direct sources of micro- and nanoplastics include the release of fibers from synthetic textiles (Cai et al., 2021) or abrasion of car tires (Järlskog et al., 2022).

Nanoplastics cannot easily be measured directly due to a lack of suitable analytical techniques (Schwaferts et al., 2019), but are assumed to be present in household dust (Zhang et al., 2020), particulate matter in outside air (Yao et al., 2022) and foodstuffs including salt (Kosuth et al., 2018), bivalves (Van Cauwenberghe and Janssen, 2014; Davidson and Dudas, 2016), fish (Kershaw and Rochman, 2015), honey (Liebezeit and Liebezeit, 2015) and water (Kosuth et al., 2018; Mason et al., 2018). Humans are therefore exposed to nanoplastics by ingestion and inhalation and subsequent health outcomes are poorly understood. Studies on human exposure to microplastic particles show that annual intake may exceed hundred thousands of particles (Cox et al., 2019; Mohamed Nor et al., 2021), while data on nanoplastic exposure is not available yet. Societal concerns on the potential health effects of micro- and nanoplastic exposure have been increasing, underpinning the need for risk assessment (Coffin et al., 2022; Felipe-Rodriguez et al., 2022).

Nanoplastics are not a homogeneous contaminant but represent a mixture of sheets, fragments and fibers of various sizes comprised mainly of polyethylene (PE), polypropylene (PP), polyethylene terephtalate (PET) and polyvinyl chloride (PVC) (https://plasticseurope.org, Karbalaei et al., 2018). This heterogeneity complicates risk assessment as past research has mostly focused on polystyrene (PS) nanospheres, which do not accurately represent the multitude of nanoplastics to which humans are exposed. Only few studies have considered alternative polymer types (Magrì et al., 2018; Busch et al., 2021b; Magrì et al., 2021; Sun et al., 2021) and even fewer studies have focused on non-spherical (Busch et al., 2022a) or weathered particles (Gopinath et al., 2021; Roursgaard et al., 2022). Despite the lack of data on prototypical environmental nanoplastics, considerable effort has been made to implement read-across methodologies based on the particles’ intrinsic properties, Mode of Action (MoA) or associated (bio)molecules, but these algorithms are not yet applicable to nanoplastics risk assessment (Walkey et al., 2014; Helma et al., 2017; Toropova and Toropov, 2022).

There is limited information available on nanoplastics toxicity *in vivo* and much of our current knowledge stems from rodent studies using microplastics. These studies need to be interpreted with caution, as some of these studies have been scrutinized because of issues related to particle characterization, (overload) exposure, detection of particles in tissues (mass balance) and methodological issues related to histopathology (Coffin et al., 2022; Gouin et al., 2022). Rodent studies using high concentrations of PS nanoplastics report bioavailability after ingestion or inhalation and particle translocation to lymph nodes, liver, spleen, and kidneys (Jani et al., 1989; Walczak et al., 2015a). More data on plastic particle toxicity is available in the context of occupational exposure to polymers (Mason, 1975; Heldaas et al., 1984; Hsiao et al., 2004). However, this information holds limited applicability to environmental nanoplastics exposure, as these studies were conducted to simulate high lung exposure concentrations in plastic industry workers (Hesterberg et al., 1992; Porter et al., 1999; Warheit et al., 2003), which is hardly comparable to the current levels of airborne plastic particles in a non-occupational environment.

Besides the direct toxicity of nanoplastics, the potential toxicity of associated chemicals and components of the eco-corona should also be considered. Nanoplastics frequently contain additives, e.g., phthalates, which may comprise up to 50% of the total mass of plastics (Andrady and Rajapakse, 2016; Hartmann et al., 2019) and often contain known toxicants like dioxins, polycyclic aromatic hydrocarbons, halogenated flame retardants and heavy metals (Bouwmeester et al., 2015; Hahladakis et al., 2018; Zimmermann et al., 2019), which could be released from the polymers after uptake (Peters et al., 2022). Furthermore, the surface of particles spontaneously adsorbs surrounding chemicals and biomolecules, leading to an eco-corona or bio-corona, respectively (Casals et al., 2010; Pulido-Reyes et al., 2017).

The plethora of possible polymers, shapes, sizes and exposure scenarios make exhaustive testing of nanoplastics a daunting task that is ethically, financially and temporally unfeasible using animal models. The acceptance of data generated in *in vitro* models for regulatory purposes is being spurred by the ban on animal testing in the cosmetics industry (Pistollato et al., 2021) and nanotoxicity testing will also have to rely on data generated *in vitro* (EFSA, 2021). Initiated by “toxicology of the 21st century” (Hartung, 2009), and to better explain MoAs, models that emulate human physiology best, should be put forward. This is boosting the development of *in vitro* models ultimately replacing animal based testing (Tannenbaum and Bennett, 2015). Currently, the main models used for *in vitro* oral or inhalation nanotoxicity testing are based on immortalized cell lines derived from cancer tissues, including Caco-2 for the intestine and A549 for the lung (Jones and Grainger, 2009; Wang M. et al., 2018; Fröhlich, 2018; EFSA, 2021). In addition to these conventional models, novel models including stem cell-based approaches have recently started being used in nanotoxicity testing. These novel methods offer advantages over cancer cell lines by having an unaltered genotype, allowing the presence of more physiologically relevant cell types and may help estimate interindividual variation. In this review, we discuss novel stem cell-based *in vitro* models of the primarily exposed organs, intestine and lung, as a new approach methodology for nanoplastics toxicity testing and how the resulting data can be used to guide future risk assessment.

## 2 Selection of *in vitro* models to study the toxicity of nanoplastics

Based on the knowledge on microplastics and engineered nanomaterials, nanoplastics mainly enter the human body through ingestion or inhalation. Therefore, these barriers are initially most at risk and studying potential toxic effects *in vitro* requires physiologically relevant models of the intestine and lung, respectively. While cell line-based models have been extensively used in toxicological research, these models lack essential features, as discussed below.

Establishing advanced barrier models requires an actively dividing and tissue-specific cell source, for example fully differentiated primary cells or somatic stem cells that can be directly isolated from the human body, often obtained from biopsies. However, the replicating ability of differentiated primary cells is limited, as differentiation generally occurs after exiting the cell cycle (Ma et al., 2019). Recently, commercially available constructs based on primary human tissue of the intestine or lung, have become available and applied for toxicological studies (Jackson et al., 2018; Janssen et al., 2021). These constructs are generated from human donors and are directly provided to the user for one-time experimental application. Although these primary models are highly relevant in terms of physiology, the user is dependent on donor availability and unobstructed delivery, as these models cannot be permanently established in a laboratory independently from the commercial provider.

In contrast to already differentiated primary tissue and cells, somatic stem cells are undifferentiated cells that divide to replenish dying cells under physiological conditions (Zakrzewski et al., 2019). These somatic stem cells, also called adult stem cells (ASCs), remain dormant until surrounding factors activate them to divide and terminally differentiate into functional cells (Urbán and Cheung, 2021). ASCs are multipotent, meaning that they can develop into more than one cell type, but they cannot produce all cell types of the body, as is the case for pluripotent cell types (Lyssiotis et al., 2011; Lairson et al., 2013). Despite improvements in long-term culture expansion of ASCs, their lifespan appears limited and inversely correlated to the donors age and highly affected by the extraction site (Bruder et al., 1997; Majors et al., 1997). Overall, the limited availability of biopsy material in combination with the finite dividing capacity of both primary cells and ASCs make them less suited to be used in toxicological studies. However, their use has greatly advanced the field of patient-specific disease modeling, i.e. cancer research and drug screening (Drost and Clevers, 2018; Driehuis et al., 2020).

Embryonic stem cells (ESCs) and induced pluripotent stem cells (iPSC), on the other hand, have gained increasing interest as a source of stem cells due to their ability to differentiate into all cell types of the organism and their high proliferative capacity (Shi et al., 2017). Human ESCs are pluripotent stem cells generally derived from the inner cell mass of blastocysts (Thomson et al., 1998). The required number of embryos used to generate the resulting ESC lines have only been reported in a small number of cases (Mehta, 2014), hinting at a low success rate, which in combination with the destructive process on the blastocyst have led to an extensive ethical debate on the use of human material (Wert and Mummery, 2003; Lo and Parham, 2009). Conversely, iPSCs do not rely on the use of embryos or invasive biopsies, forming a more ethical source of pluripotent cells. iPSCs are somatic cells (e.g. fibroblasts) reprogrammed into a pluripotent state through the forced overexpression of the four transcription factors *OCT4*, *SOX2*, *KLF4* and *MYC* (Takahashi et al., 2007). From their pluripotent state, iPSCs have been successfully differentiated to obtain tissue-specific morphology and gene expression for various organs, including the intestine (Takahashi et al., 2018) and lung (Miller et al., 2019). Embryonic-like cells derived from iPSC differentiation can be used to study a wide variety of organs and exposure scenarios, due to their proliferative capacity and the wide spectrum of available differentiation protocols (Ahmed et al., 2021). The differentiation of iPSCs into mature models of human organs usually requires multiple sequential steps in which cells are exposed to different growth factors mimicking the *in vivo* cell environment during mammalian embryonic development (Spence et al., 2011; Abud et al., 2017). As a result, the differentiation of iPSCs leads to models with a better representation of different tissue-specific cell types encountered *in vivo* (Grouls et al., 2022), in contrast to cancer lines which only represent one dominant cell type.

Particle toxicity is often evaluated based on the range of reversible and irreversible cellular damage in addition to the cells’ ability to overcome these effects, as well as signaling cascades involved in processes like proliferation and inflammation. As cancer cell lines underwent genetic changes to ensure continuous proliferation in a tumor microenvironment despite challenging metabolic conditions (Hartwell and Kastan, 1994; Sonugur and Akbulut, 2019), this could potentially lead to a skewed view on the toxicity-induced effects of nanoplastics on tissue. By contrast, stem cell-derived tissue-like cells maintain physiological proliferative behavior to provide a more representative view for toxicity-induced injury compared to cancer cell lines, while simultaneously providing multiple cell types in the same *in vitro* model. Thus, we view iPSC as a promising approach for quantitative nanoplastics toxicity testing with high physiological relevance in the primarily exposed organs intestine and lung.

### 2.1 Intestinal models

The intestine is a major part of the human digestive system, responsible for the peristalsis of chyme and the uptake of nutrients from ingested food. Accordingly, the intestinal epithelium is constantly exposed to contaminants taken up with food and water, making it an important target organ for toxicities and diseases. Therefore, research in the fields of medicine, pharmacology and toxicology has been focused on the development of suitable models to study effects on the intestine outside of animals.

To date, several studies report intestinal effects of ingested micro- and nanoplastics in a variety of *in vitro* models based on cancer cell lines. Among the available cell lines, the colorectal cancer cell line Caco-2 is by far the most utilized one (Cortés et al., 2020; Domenech et al., 2021; Stock et al., 2021), as it spontaneously differentiates into an enterocyte-like phenotype (Pinto et al., 1983), representing the most abundant cell type in the human intestine. However, several attempts have been made to increase the complexity of Caco-2 based *in vitro* models by implementing additional cell lines when investigating plastic particles (see Table 1). For example, an advanced intestinal model included HT29-MTX-E12 as goblet cells and PMA-differentiated THP-1 cells as macrophages to expand the model by the presence of mucus and an immunocompetent cell type (Busch et al., 2021a). Furthermore, a Caco-2/HT29 model supplemented with Raji-B cells to induce the differentiation into microfold cells (M-cells), a cell type specialized in transport across the epithelial barrier (Mabbott et al., 2013), was used to investigate the effects of PS nanoplastics (Domenech et al., 2020). Establishing even higher levels of complexity, a quadruple culture model was used to study plastic particle effects by complementing a Caco-2/HT29-MTX co-culture with macrophages and dendritic cells derived from human blood monocytes (Lehner et al., 2020). However, the co-culturing of different cancer cell lines and/or primary cells requires extensive fine-tuning of optimal culture conditions, e.g. in terms of medium composition, whereas different cell populations in cultures derived from stem cells originate from the same single cell source.

**TABLE 1:** Human intestinal *in vitro* models used for plastic particle toxicity assessment.

Model type		Represented tissue or cell types	References
Cell lines, monoculture	Caco-2	Intestinal epithelium (undifferentiated Caco-2) or Enterocytes (differentiated Caco-2)	Schimpel et al. (2014); Walczak et al. (2015c); Abdelkhaliq et al. (2018); Inkielewicz-Stepniak et al. (2018); Stock et al. (2019); Wu et al. (2019); Cortés et al. (2020); Liu et al. (2020); Wu et al. (2020); Domenech et al. (2021); Huang et al. (2021); Jeon et al. (2021); Stock et al. (2021); Ma et al. (2022); Steckiewicz et al. (2022); Xu et al. (2022)
	HT29	Intestinal epithelium	Inkielewicz-Stepniak et al. (2018); Visalli et al. (2021); Steckiewicz et al. (2022); Xu et al. (2022)
	LS174T	Intestinal epithelium	Inkielewicz-Stepniak et al. (2018)
	CCD 841 CoN	Intestinal epithelium	Steckiewicz et al. (2022)
	NCM 460	Intestinal epithelium	Ma et al. (2022)
	RKO	Intestinal epithelium	Xu et al. (2022)
	HIEC-6	Intestinal epithelium	Xu et al. (2022)
	HCT-116	Intestinal epithelium	Xu et al. (2022)
Cell lines, co-culture	Caco-2+HT29 (MTX)	Enterocytes and goblet cells	Mahler et al. (2012); Schimpel et al. (2014); Walczak et al. (2015b); Walczak et al. (2015c); Domenech et al. (2020); Fournier et al. (2023)
	Caco-2+Raji-B (M-cell model)	Enterocytes and microfold cells	Schimpel et al. (2014)
Cell lines, triple culture	Caco-2+HT29-MTX-E12+THP-1	Enterocytes, goblet cells and macrophages	Busch et al. (2021a); Busch et al. (2021b)
	Caco-2+HT29+Raji-B (M-cell model)	Enterocytes, goblet cells and microfold cells	Mahler et al. (2012); Schimpel et al. (2014); Walczak et al. (2015c); Domenech et al. (2020)
Cell lines, quadruple culture	Caco-2+HT29-MTX + MDDC^a^+MDM^b^	Enterocytes, goblet cells, dendritic cells and macrophages	Lehner et al. (2020)
Organoid based	Human iPSC-derived intestinal organoids	Enterocytes, Paneth cells, enteroendocrine cells and goblet cells	Hou et al. (2022)
	Human iPSC-derived colon organoids	Not reported	Park et al. (2022)
	Murine primary tissue-derived intestinal organoids	Not reported	Xie et al. (2022)

^a^ MDDC: monocyte-derived dendritic cells.

^b^ MDM: monocyte-derived macrophages.

A breakthrough toward human cell-based models with advanced physiological relevance was seen through the establishment of crypt-based intestinal organoids, enabling the long-term culture of primary intestinal cells in absence of mesenchymal tissue (Sato et al., 2009; Sato et al., 2011). Since then, research interest in stem cells has increased dramatically, resulting in the generation of numerous culture and differentiation protocols (Cao et al., 2011; Spence et al., 2011; Forster et al., 2014; Miura and Suzuki, 2017; Hou et al., 2018; Pleguezuelos-Manzano et al., 2020; Stewart et al., 2020), establishing two principal approaches for the generation of self-organized intestinal spheroids: 1) isolation of intestinal crypts and 2) use of ESCs or iPSCs.

Uniquely, these spheroids represent all major epithelial cell lineages within the organoid (Sato et al., 2011), and allow to skew them towards specific cell types and rare lineages like tuft cells (Gerbe et al., 2016; Treveil et al., 2020; Inaba et al., 2021). Even the most sophisticated non-stem cell models still fail to replicate the lineage complexity to this extent (Lozoya-Agullo et al., 2017; Lehner et al., 2020; Kämpfer et al., 2022). For the purpose of hazard testing of nanoplastics, both overall effects on the tissue as well as effects in specific individual cell types may be relevant. Using organoids, cell type-specific uptake efficiencies were demonstrated for nanoplastics (Hou et al., 2022). The authors generated intestinal organoids from an iPSC line and reported the presence of enterocytes, Paneth cells, goblet cells and endocrine cells using immunofluorescence. Uptake of 50 nm PS nanoplastics was found to be cell type-dependent, which may have been overlooked or drastically underestimated in standard culture systems, as the authors report uptake primarily in goblet, Paneth and endocrine cells. Furthermore, some cell types may be more sensitive towards stressors or noxae (Busch et al., 2022b), and might, therefore, represent important targets for exposure studies.

Also their (patho)physiological identity renders intestinal stem cell models a highly versatile tool. The cultures can replicate the complexity of various regions of the intestinal tract–from duodenum to colon–as well as healthy or diseased tissue depending on the biopsy origin or growth/differentiation protocol (Sato et al., 2011; Tsai et al., 2017; Bandi et al., 2020). In contrast, standard cell models mostly rely on cancer-derived cell lines in mono- or co-culture that often require extensive differentiation times and further manipulation to obtain rather mature cell types (Hilgers et al., 1990; Lesuffleur et al., 1990) and disease-like phenotypes (Susewind et al., 2016; Kämpfer et al., 2017). Inherently diseased, these cell lines may be of limited physiological relevance for specific research questions due to biochemical and genetic differences compared to healthy intestinal tissue, e.g. the absence or limited presence of phosphoprotein p53 (Thant et al., 2008) and cytochrome (CYP) P450 isoforms (Ohta et al., 2020). While typically considered in the context of drug metabolization (Fritz et al., 2019), CYP enzymes also play a role in the effects of xenobiotics, e.g., organic pollutants (Takeshita et al., 2011; Abass et al., 2012), which may be critical contaminants to consider in plastic particle hazard assessment (Coffin et al., 2019; Brachner et al., 2020).

It is important to also realize potential differences between stem cell-based intestinal organoids and the *in vivo* situation, for example the limited presence of mucus, although refined protocols are being developed (Navabi et al., 2013; Sontheimer-Phelps et al., 2020). Whereas human organoids express physiologically relevant types of mucins–especially mucin 2 (Sato et al., 2011)—the overall luminal coverage and thickness of the mucus remains low (VanDussen et al., 2015). In the context of particle hazard assessment, mucus is a crucial determinant for the dose of nanoparticles that can interact with the epithelium (Bandi et al., 2020).

However, stem cell-derived intestinal cultures remain scarcely used for nanoplastics hazard assessment (Lu et al., 2017; Belair et al., 2020) with only three studies on plastic particles to date (Hou et al., 2022; Park et al., 2022; Xie et al., 2022), of which two studies failed to report the differentiation procedure and/or sufficiently characterize the organoids. Even with sufficient characterization, treatment of organoids with particles is a challenge, as the organoids’ morphology–the inward-facing apical side creating a lumen–and their maintenance in semi-solid media (e.g., Matrigel) impede particle exposures. Exposures would typically occur from the apical side, which is not accessible without manipulation, while diffusion of particles is obstructed in matrices like Matrigel. Various adaptions might resolve these challenges, including luminal microinjection or application of flow-through after puncturing the organoid (Leslie Jhansi et al., 2015; Sidar et al., 2019), reversed cell polarization (i.e. “apical-out” organoids) (Co et al., 2019) or breaking the organoids’ 3D structure down to a 2D structure. Especially the latter is applied frequently, either using the whole organoid (Kasendra et al., 2018; Madden et al., 2018; Roodsant et al., 2020; Yamada and Kanda, 2021; Breau et al., 2022) or selected cell types (Workman et al., 2018; Yoshida et al., 2020). While the structural change from 3D to 2D did not substantially modify the expression of intestinal markers or functional genes (Takahashi et al., 2021), these organoid-derived cell layers can develop a high transepithelial electrical resistance (TEER>1,500 Ω•cm^2^) (Wang Y. et al., 2018), which might indicate restricted passive transport across the cell layer and may limit the transport of nanoplastics. In contrast to manipulating mature organoids back into 2D layers, other protocols are designed to differentiate iPSCs directly into intestinal epithelial monolayers, without the detour of spheroid generation (Iwao et al., 2014; Kabeya et al., 2020), resulting in more realistic TEER values of ∼250 Ω•cm^2^ (Kabeya et al., 2018). Besides 2D culturing, adaptions creating accessible luminal space or using perfusion in tubular intestinal structures are becoming increasingly sophisticated (Naumovska et al., 2020; Nikolaev et al., 2020; Wilson et al., 2021) and offer promising alternative test systems.

### 2.2 Lung models

For inhaled particles, the lung epithelium forms an initial barrier and target site of interaction. Accordingly, *in vitro* testing of particulates for lung toxicity has long used cancer or immortalized epithelial cell lines (Hiemstra et al., 2018). While being less robust and reproducible, particles have also been tested on primary epithelial cells or tissue explant cultures from lungs of rodent or human origin (Crocker et al., 1973; Craighead et al., 1980; Mohr and Emura, 1985; Driscoll et al., 1995). The introduction of stem cell-based *in vitro* models and further advancement of associated tissue engineering methodologies in pulmonary research has contributed to an improved understanding of mechanisms of lung development, damage repair and tissue regeneration processes (Gotoh et al., 2014; Basil et al., 2020). However, the use of stem cell-based models for lung toxicity testing is still in its infancy compared to, for example, developmental neurotoxicity testing (Fritsche et al., 2021; Masui et al., 2022).

In sharp contrast, the specific field of particle toxicology has witnessed crucial progress through the development of improved *in vitro* assays that use lung epithelial cell models in air liquid interface (ALI) culture conditions (Adler et al., 1990; Blank et al., 2006; Lacroix et al., 2018). Combined with aerosol generating devices, this allows for a more realistic interaction between the test particles and the surfactant-containing apical side of the epithelial cells, in contrast to models in which cells are exposed to particle suspensions under submerged conditions (Ding et al., 2020). Another innovative *in vitro* approach involves the growing of lung epithelial cells on mechanically stretchable membranes to enable testing of particles under breathing movement mimicking conditions (Schmitz et al., 2019).

To date, the pulmonary toxicity of micro- or nano-sized plastics has been tested in various *in vitro* models and aforementioned specific experimental approaches. Most investigators have either used the A549 human alveolar type II-like epithelial cell line or the BEAS-2B human bronchial epithelial cell line under conventional submerged testing conditions (see Table 2). The toxicity of PS nanoplastics has been investigated in suspension on BEAS-2B cells as well as HPAEpiC human pulmonary alveolar epithelial cells, comprised of type I and type II cells (Yang et al., 2021). A microfluidic-chip test model with BEAS-2B cells was employed by Gupta et al. (2021) to study uptake of silica particles and PS. Furthermore, A549 cells were exposed on a membrane under cyclic stretch conditions to PS nanoparticles in order to mimic the effect of breathing movements (Roshanzadeh et al., 2020). Airway and alveolar epithelial toxicity has also been explored in ALI exposure settings. For example, the uptake and inflammatory properties of various PS particles were investigated in A549 cells, as well as in the Calu-3 human upper airway cell line under ALI exposure conditions (Meindl et al., 2021). To account for the potential contribution of alveolar macrophages, the authors also included a co-culture model of A549 and THP-1 cells (Meindl et al., 2021). To the best of our knowledge, to date only one study investigating plastics has been performed using a lung organoid-based testing approach. The toxicity of polyester fibers sampled from cloth dryers was investigated in human airway organoids that were generated from tissue resident ASCs obtained from three donors (Winkler et al., 2022). Using immunofluorescence and qRT-PCR-based markers, basal cells, ciliated cells, goblet cells, club cells, and even type I cells were identified. Interestingly, the authors exposed the organoid spheres after fragmentation to allow exposure to the fibers from the outside as well as the more relevant “inner cavities” of the organoids. While the outcomes of this study may be of limited relevance in view of the dimensions of the tested materials (average length: 700 ± 400 µm and average width 10 ± 5 µm), the work provides strong proof of principle for the use of lung organoids for hazard assessment of airborne nanoplastics.

**TABLE 2:** Human lung *in vitro* models used for plastic particle toxicity assessment.

Model type	Airway model	Represented tissue or cell types	References	Alveolar model	Represented tissue or cell types	References
Cell lines, submerged	BEAS-2B	Bronchial epithelium	Lim et al. (2019); Dong et al. (2020); Gupta et al. (2021); Yang et al. (2021); Lin et al. (2022); Zhang et al. (2022b)	A549	Alveolar type II epithelial cells	Brown et al. (2001); Jeon et al. (2018); Xu et al. (2019); Zhu et al. (2020); Goodman et al. (2021); Shi et al. (2021); Bengalli et al. (2022); Florance et al. (2022); Gautam et al. (2022); Halimu et al. (2022); Shi et al. (2022); Zhang et al. (2022a); Zhang et al. (2022c); da Silva Brito et al. (2023)
				HPAEpiC	Alveolar type I and II epithelial cells	Yang et al. (2021); Zhang et al. (2022b)
Cell lines, submerged advanced	BEAS-2B in microfluidic-chip	Bronchial epithelium	Gupta et al. (2021)	A549 on membrane under cyclic stretches	Alveolar type II epithelial cells	Roshanzadeh et al. (2020)
Cell lines, ALI exposed	Calu-3	Bronchial epithelium	Meindl et al. (2021)	A549	Alveolar type II epithelial cells	Binder et al. (2021); Meindl et al. (2021)
				A549+THP-1	Alveolar type II epithelial cells and macrophages	Meindl et al. (2021)
Organoid based	Human ASC-derived airway organoid	Basal cells, ciliated cells, goblet cells, alveolar type I cells and club cells	Winkler et al. (2022)			

Similar to the intestine, a further introduction and application of stem cell-based models for pulmonary toxicity testing of nanoplastics seems promising. Herein, however, the principal advantage of exposing such models under ALI-conditions is to be emphasized in terms of dose and kinetics. Construction of complex organoid (e.g., scaffold) models, in such a way they that can be coupled to ALI exposure systems in a realistic, physiologically relevant manner, is a major challenge. If achieved, however, it will greatly contribute to improved safety evaluation of airborne nanoplastics.

## 3 Challenges and considerations when testing plastic particles *in vitro*

When studying the MoA of nanoplastics in an *in vitro* model, certain points should be carefully considered when designing the experiment, choosing relevant endpoints, or interpreting the data in regard to plastics hazard assessment:

*Low density of polymers.* Industrially produced plastics cover a whole spectrum of different densities, spanning from less than 1 g/cm³ (PP, PE), to around 1 g/cm³ (PS), to heavier polymers (PVC, polyamide (PA), PET). The density of cell culture medium is approximately 1 g/cm³, meaning that nanoplastics will either float or sediment during *in vitro* experiments, depending on their polymer composition. Polymers with a density lower than 1 g/cm³ will become buoyant when applied in a traditional cell culture system and will not establish contact with cells growing on the bottom of a well plate. So far, several approaches have been used to tackle this issue: one common approach is the inversion of the cell model, e.g. by seeding cells on cover slips that are subsequently inverted and exposed to buoyant particles from below (Stock et al., 2020), or by sealing a well plate with silicone gaskets and then inverting the entire plate (Watson et al., 2016). Furthermore, it has been reported that inverted *in vitro* models can be established by seeding cells on the basolateral side of transwell inserts (Stock et al., 2021), which also enables the application of more complex co-culture models (Busch et al., 2021b). Another approach to expose cells to buoyant plastic particles could be the use of ALI systems (Upadhyay and Palmberg, 2018). The absence of medium on the apical side of cells allows exposure to particles *via* particle-containing aerosols that sediment on the cell layers, regardless of the particles’ density. Although this approach has been used in an advanced *in vitro* model of the intestine (Lehner et al., 2020), exposure *via* aerosols is obviously more relevant for *in vitro* models representing the lung. A very specific approach to solve the buoyancy problem was introduced by Green et al. (1998). The authors embedded buoyant PE particles in the surface layer of agarose and seeded primary macrophages on top. However, due to the immobility of particles, this approach is only useful for phagocytizing cell models and not applicable for models of the intestinal or lung epithelium. A completely different approach to test buoyant polymers *in vitro* is the modification of the material to become denser. For example, mineral talc is a commonly used filler for PP to increase the materials’ performance (Ammar et al., 2017), which also increases the density of PP.

*Differences in applied dose and effective dose.* Apart from buoyant polymers, the low density of non-buoyant plastic particles needs to be considered as well. Polymers like PS are only marginally denser than cell culture medium and therefore exhibit very slow sedimentation rates. The amount of particles interacting with the cells (effective or delivered dose) is often small compared to the total amount of particles added to the *in vitro* system (applied dose). This may be further aggravated by particle agglomeration in the cell culture medium, which further decreases the density of the agglomerate (Hinderliter et al., 2010). Therefore, considerations of sedimentation kinetics for *in vitro* nanotoxicology research is important to avoid misinterpretation of concentration-dependent cellular response and uptake data (Teeguarden et al., 2007). *In silico* dosimetry models have been developed to predict the delivered dose based on the physicochemical properties of the particles and the setup of the *in vitro* exposure scenario. Currently, three main tools are used to estimate the delivered dose during *in vitro* experiments; the *In vitro* Sedimentation, Diffusion and Dosimetry model (ISDD), the *In vitro* Sedimentation, Diffusion, Dissolution and Dosimetry model (ISD3) and the Distorted Grid model (DG) (Hinderliter et al., 2010; DeLoid et al., 2015; Thomas et al., 2018). In most cases, the ISDD model is sufficient for predicting nanoplastics dosimetry. However, ISD3 and DG are better suited for modelling buoyant particles such as PE or PP, as well as polydisperse or degradable materials (Watson et al., 2016). As an example for a severe discrepancy between the applied dose and the effective dose that interacts with cells, ISDD sedimentation modelling of 50 nm PS nanoparticles showed only 17% deposition after 24 h at relevant *in vitro* conditions (Busch et al., 2021a). Contrastingly, heavier, metal-based engineered nanomaterials like silver nanoparticles of comparable size can sediment completely within 4 h under certain conditions (Kämpfer et al., 2021). Cell culture models grown under dynamic flow conditions to improve differentiation (Sargent et al., 2010) or implemented in microfluidic chips (Zhang et al., 2017; Kulthong et al., 2021) might improve the distribution of nanoplastics, increasing the correspondence between the applied and delivered dose. However, *in silico* dosimetry models are not yet equipped to predict nanoparticle sedimentation under dynamic flow conditions.

*Adherence of macromolecules.* Particles suspended in biological media like cell culture medium will acquire a biomolecular corona, i.e. proteins, lipids and carbohydrates, that adhere to the particles’ surface (Docter et al., 2015). As the adherence of these macromolecules will change the surface properties of the particles and therefore their interaction with biological systems (Monopoli et al., 2012; Cao et al., 2022), the possible role of the corona has to be considered during *in vitro* testing (Allen et al., 2006). For example, Abdelkhaliq et al. (2018) analyzed the protein corona on different PS nanoplastics *via* LC-MS/MS and reported differences based on the surface modification of the particles, which in turn impacted the uptake rates of the nanoplastics.

A physiologically relevant exposure should include the history of the particles, for example a simulated gastrointestinal digestion, or incubation in lung fluids. During *in vitro* experiments, nanoplastics are commonly applied in cell culture medium containing fetal calf serum (FCS), leading to adherence of serum proteins to the nanoplastic surface (Abdelkhaliq et al., 2018). Contrastingly, the corona of ingested nanoplastics will more likely consist of macromolecules from the food matrix and digestive fluids, while inhaled nanoplastics will exhibit a corona that may be acquired from exogenous environmental matrices (e.g. microbial components, semivolatile organic compounds) and the obvious endogenous molecules present in lung surfactant (Griese, 1999; Ichinose et al., 2005).

*Interference with test assays and readouts.* Besides changing and defining the surface identity, the adsorption of macromolecules, in particular target proteins of interest, to the surface of plastic particles, might distort the outcome of applied *in vitro* assays. Binding of extracellular lactate dehydrogenase (LDH), a common marker of membrane damage, might reduce the enzymes activity during the LDH assay, as it was reported for silver nanoparticles (Oh et al., 2014; Liang et al., 2015). Moreover, interference of amine-modified PS with the interleukin (IL)-8 ELISA was observed in the form of exaggerated IL-8 concentrations (Busch et al., 2022a). Therefore, assessing potential interferences of the tested materials with the used assays is necessary prior to toxicological investigations (Stone et al., 2009; Wilhelmi et al., 2012; Ong et al., 2014).

*Use of fluorescently labeled nanoplastics.* Often, it is important to know to what degree particles are internalized by target cells. In contrast to metal-based nanoparticles, where techniques like ICP-MS can be used to measure the cellular uptake of unlabeled particles (Mitrano et al., 2012), no equivalent techniques are currently available for polymeric particles. Instead, fluorescently labeled nanoplastics are commonly used to monitor the potency of cellular uptake, which can be quantified using confocal microscopy or flow cytometry. The use of fluorescent labels, while seemingly simple, require great care to avoid artifacts during the measurements. For instance, it is reported that fluorophores may leach out of the plastic particles, causing higher fluorescent signals in cells, or fluorescence in cell compartments not commonly available to nanoplastics (Tenuta et al., 2011; Catarino et al., 2019). Additionally, fluorophores might be pH-sensitive, leading to alterations in signal strength in subcellular compartments like lysosomes (Simonsen and Kromann, 2021), complicating interpretation of data. Another issue of using fluorescent particles is the inability to distinguish between internalization and surface adhesion of particles. Flow cytometry is commonly used to assess particle internalization, as it offers higher throughput than confocal microscopy and is more easily quantifiable. However, flow cytometry is unable to distinguish between fluorescence originating from within cells or from cell surfaces, necessitating further experiments to confirm actual internalization. This can be achieved by using dyes that are specifically quenched at the cell surface (Nuutila and Lilius, 2005; Dumont et al., 2017), by validating and correcting flow cytometry with confocal microscopy data (Gottstein et al., 2013), or by using a modified setup such as imaging flow cytometry (Phanse et al., 2012; Smirnov et al., 2015). Issues surrounding the use of fluorescence to determine nanoparticle internalization have received increased scientific interest and have been discussed in great detail in a recent review (FitzGerald and Johnston, 2021).

*Size limitations depending on the exposed organ.* Unlike engineered nanomaterials, environmental plastic particles do not have a clear size cutoff, covering a whole size spectrum from sub-nano size to several millimeters. In the case of ingested particles, size limitations only play a minor role, as even centimeter-sized plastics can be swallowed and are able to pass the human gastrointestinal tract. Therefore, the intestinal epithelium can, in theory, be exposed to particles of enormous size, justifying the testing of larger particles. For example, Lehner et al. (2020) applied microplastics up to 500 µm in an intestinal *in vitro* model. In contrast, the size of inhaled particles is strictly limited in the context of lung anatomy and physiology. Particles with an aerodynamic diameter of less than 10 µm are able to deposit in the tracheobronchial region, while only particles less than approximately 2.5 µm are small enough to reach the alveoli (Oberdorster et al., 2005; Xing et al., 2016). Therefore, when testing plastic particles in an *in vitro* model of the human respiratory system, the size of the test material has to be chosen accordingly.

*Relevance of available model particles.* When looking at available scientific literature regarding toxicological investigations of plastic particles, the majority of studies is carried out using spherical PS particles of a defined size. However, there is a strong discrepancy between available data and environmental occurrence: PS makes up only 6.1% of globally produced polymers (www.plasticseurope.org) and shapes of micro- and nanoplastics have been described to be primarily fibers, fragments and sheets (Hongprasith et al., 2020). Furthermore, plastic particles in the environment cover a whole size spectrum that might exhibit synergistic effects during co-exposure of differently sized particles (Liang et al., 2021). While PS spheres are widely commercially available, particles of other polymers or shapes are very limited. Currently, research groups mostly rely on producing their own model plastics *via* methods like laser ablation, cryocutting or milling (Magrì et al., 2018; Lionetto et al., 2021; Busch et al., 2022a), which result in much more relevant polymer types, shapes and size distributions, but might limit the comparability between different studies. The urgent need for standardized, widely available reference particles of relevant polymers, sizes and shapes has already been repeatedly expressed in recent reviews (Gouin et al., 2019; Brachner et al., 2020; Halappanavar and Mallach, 2021).

*Chemicals in nanoplastic samples.* Commercial nanoplastic suspensions are often used as model particles for the hazard assessment of plastic particles in toxicological studies. These suspensions might contain additives like preservatives, antimicrobials or surfactants, which may cause artifacts in toxicity tests. For example, the preservative sodium azide that was present in a commercial PS suspension was found to be the cause of acute toxicity towards *Daphnia magna*, instead of the PS particles themselves (Pikuda et al., 2019). Furthermore, nanoplastics produced from bulk plastic and/or plastic products might contain additives and unpolymerized monomers, like bisphenol A, heavy metals or styrene (Ajaj et al., 2021; Catrouillet et al., 2021; Gulizia et al., 2023). These chemicals might be released from the nanoplastics during incubation in the *in vitro* culture medium or in the cells that have taken up the plastic particles (Peters et al., 2022). To prevent incorrect interpretation of nanoplastics *in vitro* data and to distinguish between actual particle effects and effects stemming from associated chemicals, it is recommended to include a filtrate control in experiments (Petersen et al., 2022).

## 4 Conclusion and future outlook

Currently developed (advanced) *in vitro* models, due to their increased physiological relevance for human hazard assessment and suitability for in-depth MoA studies, are well positioned for contributing to solve the complex issues surrounding nanotoxicity. Different *in vitro* models are suited to tackle different challenges and depending on the research question, stem cell-based models might be a suitable approach for the investigation of nanoplastics uptake and effects in the primarily exposed organs intestine and lung, as they offer specific advantages over traditional immortalized cell lines.

Common knowledge derived from *in vitro* models includes the prediction of absorption rates, the derivation of MoAs and the estimation of potency by relating *in vitro* endpoints to *in vivo* endpoints (Bernauer et al., 2005). The derivation of MoAs highly depends on the physiological relevance of the model and partly on the cell types present. Conventional cell models benefit from a high relative robustness compared to stem cell models and are comparatively easy to culture, which may lead to a higher interlaboratory comparability and more accurate estimation of the benchmark dose (Black et al., 2022) when comparing different nanoparticles. This is especially true when comparing results found in different stem cell-based models, where a lack of consistency could imaginably complicate risk assessment. However, in the case of cellular processes altered due to the disease status of cancer cell lines, including genotoxicity, proliferation and cytokine production/response (Kauffman et al., 2013), iPSCs may be more relevant to study the real world situation. Similarly, when considering omics-based MoA screening approaches, the multitude of mutations commonly seen in cancer cell lines (Hartung and Daston, 2009) can alter RNA or protein expression and can mask the true effect of many small molecule compounds (Ben-David et al., 2018) and potentially also nanoplastics. Especially when using approaches such as systems toxicology (Hartung et al., 2012), small alterations in e.g. transcriptomics might help identifying important early signaling events at low exposure concentrations that might only lead to detectable toxicity at prolonged or repeated exposure. Furthermore, while the lack of robustness of stem cell-based models can be seen as a demerit, these models do allow for the estimation of relative sensitivity of subpopulations of cells or individuals with a particular genetic background. Such information can be especially relevant for risk assessment as this reduces the need for potentially inaccurate scaling factors. While *in vivo* studies are commonly used to derive the lower confidence limit of the benchmark dose (BMDL) and organ concentrations, special care must be taken when interpreting these results. The saying “mice are not men” (Warren et al., 2015) especially holds true regarding nanoparticles where differences in physical barriers including the amount of Peyers patches and M-cells, as well as the structure of the mucus layer in intestine and lung can cause further discrepancies between the human and animal situation (Kararli, 1995; Fagerholm et al., 1996; Ermund et al., 2013).

While the methodologies to scale *in vitro* nanoparticle toxicokinetics to *in vivo* kinetics are in the explorative phase, they are the only conceivable, consistent source of human toxicokinetic data. When considering *in vitro* models for the possibility of extrapolation from *in vitro* to *in vivo* (i.e. quantitative *in vitro* to *in vivo* extrapolations or QIVIVE), stem cell-based models seem to be favored as these consist of non-diseased tissue made up of the relevant cell types found *in vivo*. In view of replacing animal experiments, efforts have already been made to apply physiologically based kinetic (PBK) models to nanomaterials in order to extrapolate *in vitro* uptake data to the *in vivo* situation (Brouwer et al., 2023). Although the acceptance of PBK model data in a regulatory context is still limited (Lamon et al., 2019), using input data from physiologically relevant stem cell-based *in vitro* models might facilitate the development in this direction.

The identification of key events leading to adverse health outcomes and subsequent implementation in an adverse-outcome-pathway (AOP) frame is currently explored for nanomaterials. These AOPs, and associated key events, might aid in selecting relevant *in vitro* assays. It is expected that commonly used *in vitro* assays can be used, however some emphasis to typical “nano”-related endpoints are foreseen, such as cellular uptake, membrane damage, ROS generation or release of pro-inflammatory cytokines (Jagiello and Ciura, 2022). However, the molecular initiating events (MIE) of nanoparticles leading to toxicity are often unspecific, in direct contrast to the MIE of a chemical that binds to a receptor or target molecule (Halappanavar et al., 2020). Therefore, translation of *in vitro* effects to the *in vivo* situation in the case of nanomaterials including nanoplastics still requires extensive development, but will greatly benefit from *in vitro* models that closely mimic the *in vivo* situation, such as the stem cell-based models discussed in this review.

Despite the unique features that qualify stem cell-based models as a promising approach for future nanotoxicity studies, there are some limitations that can make the use of these models challenging. The cultivation and differentiation of stem cells into a mature model usually covers time spans of two weeks or more for a single experiment (Kabeya et al., 2018; Naumovska et al., 2020), and is quite expensive in terms of consumption of medium and the multitude of growth factors required for differentiation. The handling of stem cells requires highly trained operators, since wrong handling can easily lead to death or spontaneous differentiation of stem cells, e.g., by suboptimal cell confluence or inappropriate passaging (Kogut et al., 2014; Castro-Viñuelas et al., 2021; Yamamoto et al., 2022), which drastically impairs the robustness of the model itself. This issue of robustness is further supported by the lack of standardized protocols for stem cell-based models in the current literature.

Another current limitation of both cell line and stem cell-based models is the lack of immunocompetent cells, as immune cells like macrophages or neutrophils are derived from hematopoietic stem cells in the bone marrow, instead of tissue-resident stem cells, and transported *via* the blood (Epelman et al., 2014; Leiding, 2017). Organ models based on stem cells exclusively consist of the cell types that stem from the respective tissue-resident stem cells. Yet, pro-inflammatory effects appear to be one of the main mechanisms of nanoplastics toxicity, as they have been described in several *in vivo* studies (Choi et al., 2021; Sun et al., 2021; He et al., 2022; Tang et al., 2022). Similarly, nanoplastics have been shown to interact with components of the nervous system (Jung et al., 2020), which stem cell-based models of intestine and lung are currently lacking. However, the generation of immune and neural cells from iPSCs has been reported (McKinney, 2017; Trump et al., 2019; Gutbier et al., 2020) and the co-culture of mature stem cell-based models with additional cells is possible, e.g. intestinal organoids with an enteric nervous system (Noel et al., 2017; Takahashi et al., 2017; Workman et al., 2017; de Rus Jacquet, 2019; Choi et al., 2022). However this has, to the best of our knowledge, not been applied in the field of particle toxicology yet.

The limitations of stem cell-based models, such as the reduced robustness and the lack of standardized protocols might eventually be overcome, as the field develops rapidly and new insights are gained frequently. Similarly, the implementation of other relevant cell types into these models will be the next step towards highly versatile alternatives to animal testing, as it will be possible to investigate immune- or nervous system-related effects of nanoplastics in a physiologically relevant *in vitro* environment.

Lastly, recreating dynamic conditions as present in the intestine and lung by creating *in vitro* models in microfluidic devices (i.e. organ-on-a-chip) might also help to tackle some specific particle-related challenges such as the buoyancy issue. Initial work using a microfluidic-chip model has been performed by exposing human bronchial BEAS-2B cells to PS (Gupta et al., 2021). By generating scaffold-like (Shim et al., 2017) or tubular structures (Naumovska et al., 2020) inside of microfluidic devices that are exposed to a dynamic flow of particle suspension, the sedimentation behavior of buoyant particles will no longer be an obstacle when testing polymers like PP or PE, allowing more accurate dosimetry estimates in intestinal or non-ALI lung models. The adaption of protocols and settings of the microfluidic devices for the purpose of particle testing will require extensive optimization, such as flow rates, exposure time and exact dosimetry calculations. However, the successful integration of such advanced models into the portfolio of validated methods of *in vitro* nanotoxicology will greatly enhance the field.
